# A New Thermodynamic Model to Approximate Properties of Subcritical Liquids

**DOI:** 10.3390/e25071002

**Published:** 2023-06-29

**Authors:** Susana Sánchez-Orgaz, Fernando Varela, Javier Rodríguez, Celina González

**Affiliations:** 1 Department of Energy Engineering, Universidad Politécnica de Madrid, 28006 Madrid, Spain; javier.rodriguez.martin@upm.es (J.R.); celina.gonzalez@upm.es (C.G.); 2 Department of Energy Engineering, Universidad Nacional de Educación a Distancia (UNED), 28040 Madrid, Spain; fvarela@ind.uned.es

**Keywords:** classical thermodynamics, incompressible substance models

## Abstract

In order to obtain the thermodynamic properties of compressed liquids, it is usual to consider them as incompressible systems, since liquids and solids are well represented by this thermodynamic model. Within this model, there are two usual hypotheses that can be derived in two different submodels: the strictly incompressible (SI) model, which supposes a constant specific volume $v=v_{0}$, and a more general model, called temperature-dependent incompressible (TDI) model, which relates a specific volume to temperature, $v=v\left(T\right)$. But, usually, this difference ends here in the thermal equation of state, and only the SI model was developed for caloric and entropic equations. The aim of this work is to provide a complete formulation for the TDI model and show where it can be advantageously used rather than the SI model. The study concludes that the proposed model outperforms the traditional model in the study of subcritical liquid. One conceivable utilization of this model is its integration into certain thermodynamic calculation software packages (e.g., EES), which integrate the more elementary SI model into its code for certain incompressible substances.

## 1. Introduction

It is a frequent occurrence for substances in a liquid or solid state to manifest in the analysis of energy systems, such as thermal oils, molten salts, and liquid refrigerants. In this regard, it is noteworthy to mention that molten salts and thermal oil are used as media for thermal energy storage and cycle fluid in solar power plants [1,2,3,4].

In order to perform energy and entropy/exergy balances for systems involving these particular media, it becomes imperative to ascertain their thermodynamic properties, notably encompassing internal energy, enthalpy, and entropy.

A commonly employed approach for such substances involves the utilization of a straightforward, yet satisfactory, thermodynamic model known as the incompressible substance model [5,6,7]. Within this framework, either a constant specific volume (SI model) or a dependence solely on temperature (TDI model) is assumed, which significantly simplifies the formulation process and facilitates the derivation of these properties by leveraging saturation data, which are typically more readily available, or a specific heat capacity along with corresponding pressure and temperature values.

The thermal equation of state relates volume to temperature and pressure.

$$v=v\left(T,P\right)$$

or, in differential form,

$$dv=v\beta dT-vk_{T}dP$$

where β is the coefficient of volume expansion, and $k_{T}$ is the isothermal compressibility coefficient,

$$\beta =\frac{1}{v}{\left.\frac{\partial v}{\partial T}\right|}_{P}$$

$$k_{T}=-\frac{1}{v}{\left.\frac{\partial v}{\partial P}\right|}_{T}$$

So far, the general form of an equation of state, understanding that their precision and validity range will depend on the quantum mechanical model, has been applied to describe the behavior of substances at the microscopic level and that will be reflected in the parameters or coefficients of the equations of state.

In this point, nearly every consulted paper on engineering thermodynamics [5,6,7] make an aside commenting on some particular substances whose volume does not essentially depend on pressure and depends a very little bit on temperature. Those substances are called *incompressible substances*. Their description combines the behaviors of liquids and solids; however, this article is only concerned with liquids. 

If precision is needed for the calculation of properties of liquid substances (pure or mixtures), general equations of state are used that incorporate semi-empirical models in the calculation of the characteristic parameters of the equation. 

The modified Rakket equation [8] has been mostly used to calculate the volume of liquids, but nowadays, cubic equations, especially Soave and Peng Robinson ones, are preferred. Peneloux [9] used the latter to publish a volume of 233 substances with a global deviation of 5.2%. Improvements in the accuracy of the cubic equations of state have continued to be made, resulting in very small overall errors in the calculation of the volume because they introduce two or more parameters characteristic of the substances, which complicates the calculation considerably.

There are many cases in which additional hypotheses can be made for the formulation of the general equations of state, $k_{T}=0$ (null dependence of volume with pressure) and, sometimes, β=0 (no dependence of volume on temperature).

Depending on whether only the first hypothesis is applied, or both, the thermal equation of state remains, respectively:

dv=vβdT

or

dv=0

This is, $v=v\left(T\right)$ for the first model named the *temperature-dependent incompressible substance model* (TDI) and v=const. for the second model called *strictly incompressible substance model* (SI).

In most of the energy installations in which pumps are used, the most used model for the working fluid is the SI model [10,11]; this model is also incorporated into the widely used software, Engineering Equation Solver (EES). 

When the first principle is analyzed, caloric equations $u=u\left(T,v\right)$ and $h=h\left(T,P\right)$ arise, and when incompressible substance models were applied to these equations, only the SI model was developed [12], resulting in

du=cdT

and

dh=cdT+vdP

where $c\left(T\right)=c_{v}\left(T\right)=c_{p}\left(T\right)$ is the specific thermal capacity of the substance, which is dependent only on the temperature.

The second principle for incompressible substances is also described only through the SI model, leading us to the expression for entropy.

$$ds=\frac{c}{T}dT$$

This paper proposes a new model called TDI for the calculation of thermodynamic properties: specific internal energy (u), specific enthalpy (h) and specific entropy (s), of liquids in a simple way. The results were analyzed by comparing them with the ones of the *strictly incompressible substance model* (SI) model, which is widely used, calculating the errors of both with respect to a chosen equation of state. For this purpose, three different fluids were studied, for which the aim was to find areas where the new model was applicable and whether improvements could be obtained using the SI model.

It is shown that this model presents some advantages over the commonly used SI model for subcritical liquids: better accuracy, simplicity, and low-cost implementation using thermodynamical software tools. 

## 2. Materials and Methods

### 2.1. Development of the Model

The starting point of the model is the thermal equation of state, where the specific volume is independent of the pressure:

$$v=v\left(T\right),$$

Or, in terms of differentials,

$$dv=v^{\prime }\left(T\right)dT=v\cdot \beta \left(T\right)dT$$

#### 2.1.1. Caloric Equations

As v, T are dependent properties, we must choose $\left(T,\;P\right)$ as variables of state. So, the proper caloric equation of state is $h=h\left(T,P\right)$, for which the differential form is

$$dh=c_{p}dT+{\left.\frac{\partial h}{\partial P}\right|}_{T}dP$$

With the aid of the second principle and some thermodynamic relations [6], it finally can be written as

$$dh=c_{p}dT+\left(v-T{\left.\frac{\partial v}{\partial T}\right|}_{P}\right)dP$$

and using the thermal equation of state,

$${\left.\frac{\partial v}{\partial T}\right|}_{P}=v^{\prime }\left(T\right)=\beta v$$

which leads to

(1) $$dh=c_{p}dT+v\left(1-T\beta \right)dP$$

and since

du=dh−Pdv−vdP

(2) $$du=c_{p}dT-T\cdot v^{\prime }\left(T\right)dP-Pv^{\prime }\left(T\right)dT=\left(c_{p}-P\beta v\right)dT-T\beta vdP$$

The first thing that can be observed is that, in contrast with the SI model, specific internal energy, u, is a function of pressure, $u=u\left(T,P\right)$. 

Enthalpy Equation (1) is a corrected version of strictly incompressible equation, which is

dh=cdT+vdP

The pressure dependence of enthalpy is modified by subtracting a term, Tβv, over the specific volume, v. The specific volume usually increases with temperature, β>0, and dependence of enthalpy on pressure diminishes in this model with respect to that of the SI model.

#### 2.1.2. Constant Pressure Specific Thermal Capacity

The state function $c_{p}\left(T,P\right)$ fulfills the following equation for any system [6]:

$${\left.\frac{\partial c_{p}}{\partial P}\right|}_{T}=-T{\left.\frac{\partial^{2}v}{\partial T^{2}}\right|}_{P}$$

In particular, for our model,

$${\left.\frac{\partial c_{p}}{\partial P}\right|}_{T}=-T\cdot v\prime \prime \left(T\right)$$

Which allows us to compute $c_{p}\left(T,P\right)$ for any pressure, P, when we know the function for a determined reference pressure, $P_{ref}$:

(3) $$c_{p}\left(T,P\right)=c_{p}\left(T,P_{ref}\right)+\int_{P_{ref}}^{P}{\left.\frac{\partial c_{p}}{\partial P}\right|}_{T}dP=c_{p}\left(T,P_{ref}\right)-T\cdot v\prime \prime \left(T\right)\left(P-P_{ref}\right)$$

In the case of incompressible fluids, liquids, and solids, it is common to have the tabulated density and $c_{p}$ data for $P_{ref}=1\;\mathrm{atm}$ for the function of temperature. 

#### 2.1.3. Entropy Equations

Again, and for the previously given reasons, we chose $\left(T,\;P\right)$ as the variables of state. The second Tds equation

Tds=dh−vdP 

along with some generalized thermodynamic relationships [6] lead to

$$ds=\frac{c_{p}}{T}dT-{\left.\frac{\partial v}{\partial T}\right|}_{P}dP\;$$

In our concrete case, as $v=v\left(T\right)$,

(4) $$ds=\frac{c_{p}}{T}dT-v^{\prime }\left(T\right)dP=\frac{c_{p}}{T}dT-\beta vdP\;$$

Which also adds some dependency of the entropy, which pressure respect to the SI model, which is

$$ds=\frac{c}{T}dT\;$$

Again, the dependence of entropy on pressure diminishes in this model.

### 2.2. Calculation of Properties According to the Model

At this point, equations of state in differential forms have been given. However, to obtain values of ∆u, ∆h and ∆s, Equations (1), (2) and (4) must be integrated.

It is not difficult to perform these integrations, but at the same time, they are not straightforward since they cannot be conducted using separate variables. As we observed, potential functions in $\left(v,\;P\right)$ of the differential forms must be calculated first.

#### 2.2.1. Specific Enthalpy 

The differential form for enthalpy is

$$dh=c_{p}dT+\left(v-T\cdot v^{\prime }\left(T\right)\right)dP\;$$

It is easy to check that the differential form is exact, i.e., it fulfills Schwartz’s relationship for integrability, since

$${\left.\frac{\partial c_{p}}{\partial P}\right|}_{T}=-T\cdot v\prime \prime \left(T\right)={\left.\frac{\partial }{\partial T}\right|}_{P}\left(v-T\cdot v\prime \left(T\right)\right)\;$$

It is not difficult either to check that the difference of enthalpies, once the exact differential form (1) has been integrated, can be calculated as

(5) $$\begin{array}{cc} ∆h & =\int_{T_{1}}^{T_{2}}c_{p}\left(T,P_{ref}\right)dT+{\left[\left(P-P_{ref}\right)\cdot \left(v-T\cdot v^{\prime }\left(T\right)\right)\right]}_{\left(T_{1},P_{1}\right)}^{\left(T_{2},P_{2}\right)}\\ & =\int_{T_{1}}^{T_{2}}c_{p}\left(T,P_{ref}\right)dT+{\left[\left(P-P_{ref}\right)\cdot v\left(1-T\beta \right)\right]}_{\left(T_{1},P_{1}\right)}^{\left(T_{2},P_{2}\right)}\; \end{array}$$

#### 2.2.2. Specific Internal Energy

Since $∆u=∆h-∆\left(Pv\right)$, the TDI model for internal energy is:

$$∆u=\int_{T_{1}}^{T_{2}}c_{p}\left(T,P_{ref}\right)dT+{\left[\left(P-P_{ref}\right)\cdot \left(v-T\cdot v^{\prime }\left(T\right)\right)\right]}_{\left(T_{1},P_{1}\right)}^{\left(T_{2},P_{2}\right)}-∆\left(Pv\right)\;$$

(6) $$\begin{array}{cc} ∆u & =\int_{T_{1}}^{T_{2}}c_{p}\left(T,P_{ref}\right)dT-∆v\cdot P_{ref}-{\left[T\cdot v^{\prime }\left(T\right)\left(P-P_{ref}\right)\right]}_{\left(T_{1},P_{1}\right)}^{\left(T_{2},P_{2}\right)}\\ & =\int_{T_{1}}^{T_{2}}c_{p}\left(T,P_{ref}\right)dT-∆v\cdot P_{ref}-{\left[T\beta v\left(P-P_{ref}\right)\right]}_{\left(T_{1},P_{1}\right)}^{\left(T_{2},P_{2}\right)}\; \end{array}$$

#### 2.2.3. Specific Entropy

Differential form for entropy in TDI model is:

$$ds=\frac{c_{p}}{T}dT-v^{\prime }\left(T\right)dP\;$$

which is exact since it comes from a state function and integration:

(7) $$\begin{array}{cc} ∆s & =\int_{T_{1}}^{T_{2}}\frac{c_{p}}{T}\left(T,P_{ref}\right)dT-{\left[\left(P-P_{ref}\right)\cdot \left(v^{\prime }\left(T\right)\right)\right]}_{\left(T_{1},P_{1}\right)}^{\left(T_{2},P_{2}\right)}\\ & =\int_{T_{1}}^{T_{2}}\frac{c_{p}}{T}\left(T,P_{ref}\right)dT-{\left[\left(P-P_{ref}\right)\beta v\right]}_{\left(T_{1},P_{1}\right)}^{\left(T_{2},P_{2}\right)} \end{array}$$

### 2.3. Applications of the Model

The TDI model can be used advantageously mainly in two situations:

1. For the approximation of compressed liquid property values when saturation values are known.
2. For the calculation of the specific internal energy $\left(u\right)$, specific enthalpy $\left(h\right)$ and specific entropy $\left(s\right)$ of any incompressible system with data of specific volume and specific thermal capacity at a reference pressure as a function of temperature.

#### 2.3.1. Approximation of Compressed Liquid Properties from Saturation

We can approximate the compressed liquid property values from saturation values at the same temperature by choosing two points that lay in the same isotherm, T, using (5)–(7),

$$∆h=∆P\cdot v\left(1-\beta T\right)$$

∆u=−Tβv·∆P

∆s=−∆P·βv

**Figure 1:**
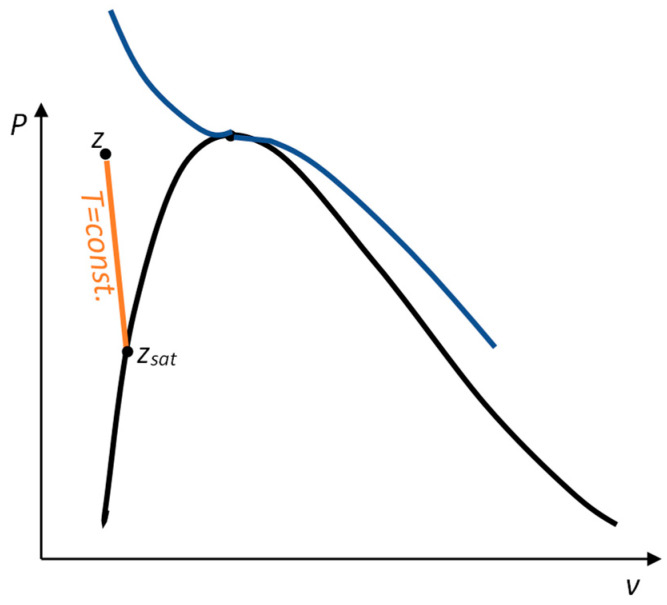
Approximating compressed liquid property values with saturation states.

For one of these points, the saturation points has the same temperature as the desired point, as seen in Figure 1.

We have the expressions:

$$h-h_{sat}=\left(P-P_{sat}\right)\cdot v_{sat}\left(1-T\beta_{sat}\right)$$

$$u-u_{sat}=-T\beta_{sat}v_{sat}\left(P-P_{sat}\right)$$

$$s-s_{sat}=-\left(P-P_{sat}\right)\cdot \beta_{sat}v_{sat}$$

which correct the expressions for SI fluids:

$$h-h_{sat}=\left(P-P_{sat}\right)\cdot v_{sat}$$

$$u-u_{sat}=0$$

$$s-s_{sat}=0$$

The difference between these two sets of equations are the correction terms:

(8) $$-\left(P-P_{sat}\right)\cdot Tv\prime_{sat}$$

for enthalpy and internal energy, and

(9) $$-\left(P-P_{sat}\right)\cdot v\prime_{sat}$$

for entropy.

Thus, the utility of this TDI equation in comparison with that of the correspondent SI arises when these correction values are high, i.e., the pressures are much greater than the saturation pressure is, $P\gg P_{sat}$ (for u,h,s) and/or temperatures are, T↑↑ (for u,h).

For ${v^{\prime }}_{sat}$ calculation, we can consider the interpolation and derivation of $v_{sat}$ values or numerical derivation, e.g., a centered finite difference using near-saturation-specific volume values:

(10) $${v^{\prime }}_{sat}\left(T\right)≅\frac{v_{sat}\left(T+∆T\right)-v_{sat}\left(T-∆T\right)}{2\cdot ∆T}$$

#### 2.3.2. Calculation of u,h,s of an Incompressible System

Suppose we have some tabulated data of a specific volume and a specific thermal capacity for a given pressure (Table 1).

Then, using Equations (5)–(7), either using interpolation functions or quadrature rules for integration and numerical derivations and setting origins $u\left(T_{0},P_{0}\right)=0,\;s\left(T_{1},P_{1}\right)=0$ (origin for h must be derived from $u\left(T_{0},P_{0}\right)$ if we want compatibility in energy balances), we can obtain

$$u=\int_{T_{0}}^{T}c_{p}\left(T,P_{ref}\right)dT-\left(v\left(T\right)-v\left(T_{0}\right)\right)\cdot P_{ref}-{\left[T\cdot v^{\prime }\left(T\right)\left(P-P_{ref}\right)\right]}_{\left(T_{0},P_{0}\right)}^{\left(T,P\right)}$$

$$h=P_{0}\cdot v\left(T_{0},P_{0}\right)+\int_{T_{0}}^{T}c_{p}\left(T,P_{ref}\right)dT+{\left[\left(P-P_{ref}\right)\cdot \left(v-T\cdot v^{\prime }\left(T\right)\right)\right]}_{\left(T_{0},P_{0}\right)}^{\left(T,P\right)}$$

$$s=\int_{T_{1}}^{T}\frac{c_{p}}{T}\left(T,P_{ref}\right)dT-{\left[\left(P-P_{ref}\right)\cdot \left(v^{\prime }\left(T\right)\right)\right]}_{\left(T_{1},P_{1}\right)}^{\left(T,P\right)}$$

The integral terms

$$\int_{T_{0}}^{T}c_{p}\left(T,P_{ref}\right)dT;\int_{T_{1}}^{T}\frac{c_{p}}{T}\left(T,P_{ref}\right)dT$$

can be calculated by using a quadrature rule (trapezoidal rule, Simpson rule, etc.) when one has discrete values of $c_{p}\left(T,P_{ref}\right)$ or directly integrating the expression if available (fitting curve). The value of $v^{\prime }\left(T\right)$ can be calculated as shown in Equation (10).

## 3. Results and Discussion

To check the proposed model, some compressed liquid states were considered, and we chose water, CO_2_, isobutane and R134A as test substances. The selection of these fluids was made due to their common use in the industry, particularly in the fields of power generation and refrigeration.

The relative errors of the TDI and SI models are shown for the state functions, u, h and s, which are defined as:

$$\varepsilon_{TDI}=\frac{\left|z_{TDI}-z\right|}{z}\cdot 100$$

$$\varepsilon_{SI}=\frac{\left|z_{SI}-z\right|}{z}\cdot 100$$

where z is the *real* value of the property obtained using the thermodynamic property calculation software *Coolprop* [13] and $z_{TDI},\;z_{SI}$ the TDI and SI approximations. *Coolprop* equations for incompressible substances can be found in [14].

Test cases were chosen fixing a reduced temperature, $T_{r}=\frac{T}{T_{crit}},$ common for the different substances, such that temperature in all the substances is above the triple point, $T_{3}$, and below the critical one:

$$T_{3}/T_{crit}<T_{r}<1,$$

and pressures above saturation for that temperature, $P>P_{sat}\left(T\right),$ up to the critical pressure, i.e.,

$$P_{sat}\left(T\right)<P<P_{crit}$$

The decimal logarithmic error comparison of both methods is presented below for four test substances and for a set of reduced temperatures in the interval defined above for specific internal energy, specific enthalpy, and specific entropy.

The data obtained in the case of water are discussed below; the rest of the data and graphs can be found in Appendix A.

**Figure 2:**
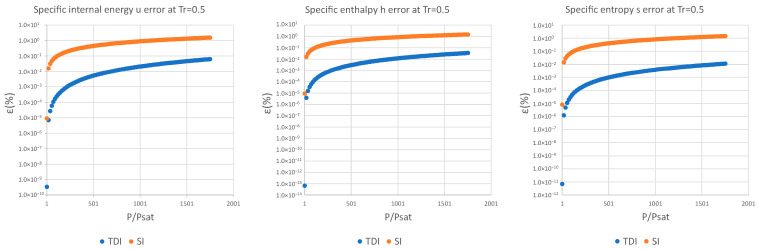
Relative errors for TDI and SI models in u,h,s at $T_{r}=0.5$.

**Figure 3:**
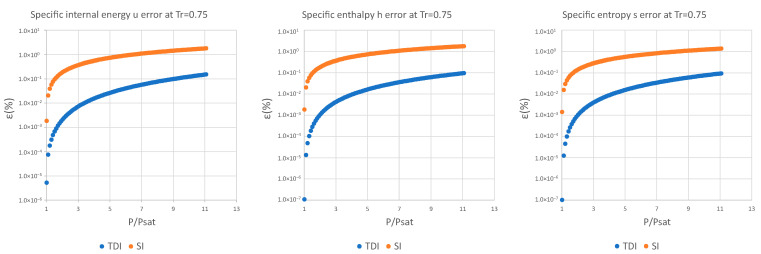
Relative errors for TDI and SI models in u,h,s at $T_{r}=0.75$.

**Figure 4:**
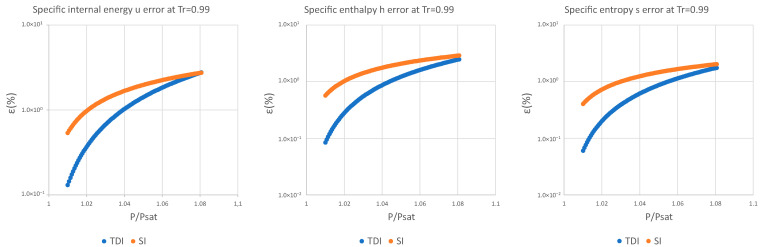
Relative errors for TDI and SI models in u,h,s at $T_{r}=0.99$.

It can be observed in Figure 2, Figure 3 and Figure 4 that for the test substance, water, on the same isotherm, the relative error of both methods increased as we moved away from the saturation pressure, with the TDI method being more accurate than the SI method was up to the critical pressure. This behavior extends to the other substances studied (see figures in Appendix A).

The following table gives the numerical values of the relative errors for the TDI model for water as a function of reduced temperature within the pressure range defined above, Table 2.

As can be seen in the above figures and in the figures in Appendix A, the behavior of the model is essentially the same as those for the four test substances at the same reduced temperature. Moreover, this behavior is essentially the same for the three studied properties: fixed a reduced temperature, both errors grew with pressure above the saturation.

In numerical terms of relative error, the specific internal energy, u, behaves worse (one/two orders of magnitude less) than enthalpy and entropy do, where the methods have a similar performance: for a reduced temperature $T_{r}=0.5$, the minimum relative errors, $\varepsilon_{TDI,\;min},$ are around $10^{-12}$ in h,s while for u they are around $10^{-10}$ for $T_{r}=0.75$, $\varepsilon_{min,\;u}\approx 10^{-6}$ and $\varepsilon_{min,\;h,s}\approx 10^{-7}$. For maximum errors, the same trend is observed.

**Figure 5:**
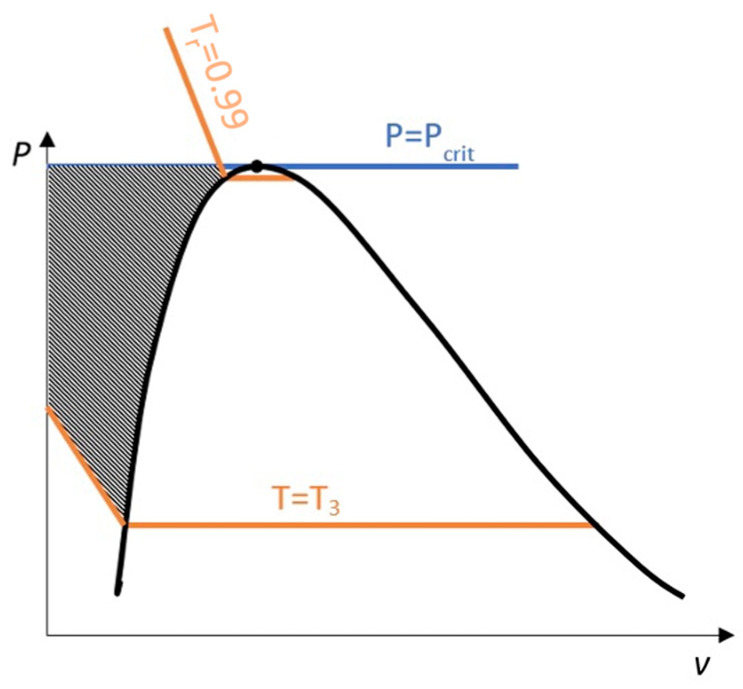
Usage region for TDI model.

Near saturation pressure, the TDI model behaves much better than the SI model does, as expected, but as the pressure grows, the SI model performance becomes more similar to that of the TDI model and finally surpasses it at around $T_{r}=0.99$. This practically fixes the usage region for the TDI model as all the subcritical liquid zone ($P\leq 0.99\cdot P_{crit}$) where the TDI model has better accuracy than the SI model does (Figure 5).

## 4. Conclusions

A complete thermodynamic model for temperature-dependent incompressible fluids (TDI) was developed from the thermal equation of state, $v=v\left(T\right),$ and the generalized relationships among thermodynamic properties.

Approximations for compressed liquids from isothermal saturation points were deduced for this model and compared to those of the classic thermodynamic incompressible model, v=const. (SI).

Both models were compared to real values in this context, obtaining relative errors for state functions, u,h,s.

When approximating sub-critical compressed liquid properties from saturation states, the TDI model presents a better behavior than SI model does; this behavior improves when the pressure tends to saturation pressure.

As the equations of the model show (Equations (8)–(10)), 4 extra summation/subtraction and 3/4 multiplications per property are needed for the SI model. Hence, it is expected that the practical implementation of this model in software applications such as EES or similar ones will not entail a significantly higher computational cost.

## Figures and Tables

**Table 1:** Generic incompressible system data.

T	v	$c_{p}$
$T_{1}$	$v_{1}$	$c_{p1}$
$T_{2}$	$v_{2}$	$c_{p2}$
$T_{3}$	$v_{3}$	$c_{p3}$
…	…	…

**Table 2:** Relative errors for TDI model for water.

$T_{r}$	$\varepsilon_{TDI.\;min.u}$ (%)	$\varepsilon_{TDI.\;max.u}$ (%)	$\varepsilon_{TDI.\;min.h}$ (%)	$\varepsilon_{TDI.\;max.h}$ (%)	$\varepsilon_{TDI.\;min.s}$ (%)	$\varepsilon_{TDI.\;max.s}$ (%)
0.45	9.98 × 10^−11^	2.11 × 10^−13^	6.32 × 10^−11^	2.42 × 10^−3^	2.24 × 10^−1^	1.38 × 10^−1^
0.5	3.46 × 10^−10^	6.90 × 10^−14^	7.02 × 10^−12^	6.26 × 10^−2^	3.50 × 10^−2^	1.15 × 10^−2^
0.6	3.33 × 10^−8^	1.63 × 10^−10^	2.72 × 10^−10^	7.79 × 10^−2^	2.34 × 10^−2^	4.33 × 10^−2^
0.75	5.23 × 10^−6^	1.09 × 10^−7^	1.03 × 10^−7^	1.52 × 10^−1^	9.65 × 10^−2^	9.42 × 10^−2^
0.9	4.26 × 10^−4^	3.41 × 10^−5^	2.59 × 10^−5^	5.05 × 10^−1^	4.05 × 10^−1^	3.12 × 10^−1^
0.99	1.31 × 10^−1^	8.45 × 10^−2^	6.04 × 10^−2^	2.76 × 10^0^	2.48 × 10^0^	1.77 × 10^0^

**Figure A1:**
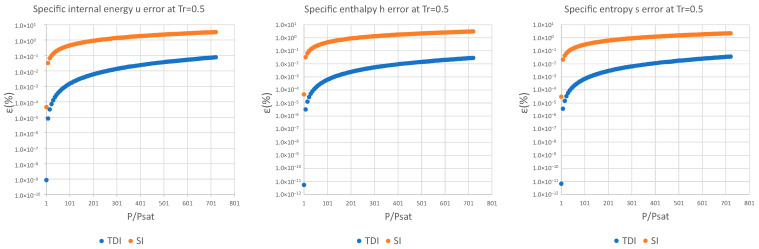
Relative errors for TDI and SI models in u,h,s at $T_{r}=0.5$.

**Figure A2:**
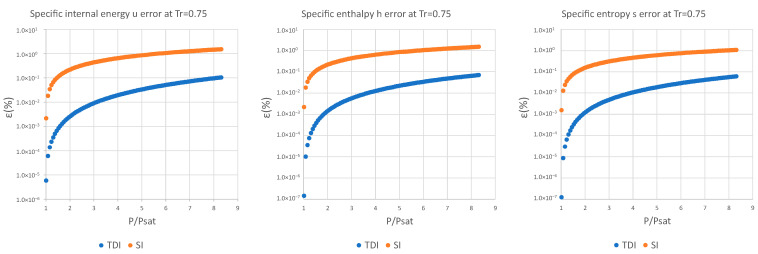
Relative errors for TDI and SI models in u,h,s at $T_{r}=0.75$.

**Figure A3:**
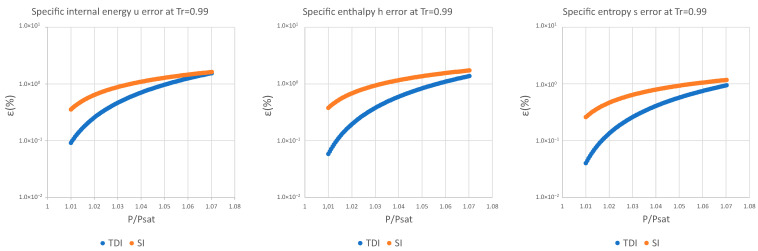
Relative errors for TDI and SI models in u,h,s at $T_{r}=0.99$.

**Figure A4:**
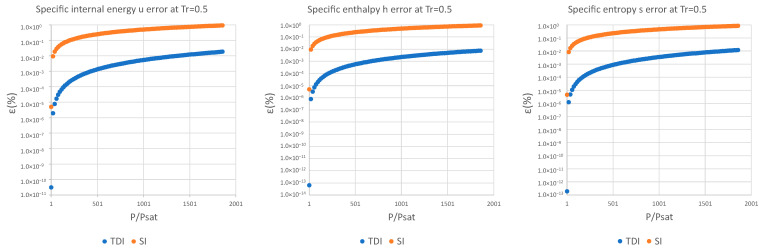
Relative errors for TDI and SI models in u,h,s at $T_{r}=0.5$.

**Figure A5:**
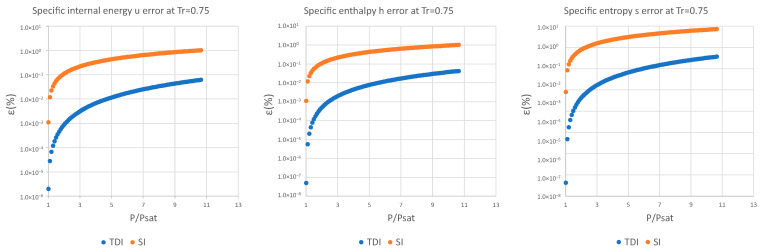
Relative errors for TDI and SI models in u,h,s at $T_{r}=0.75$.

**Figure A6:**
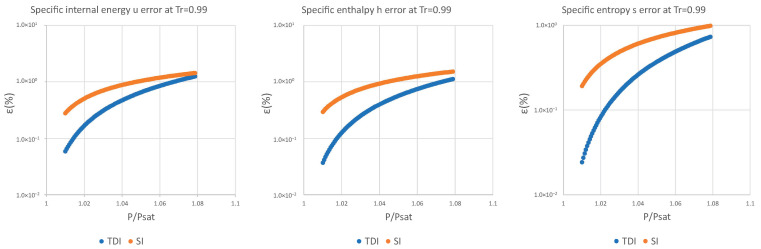
Relative errors for TDI and SI models in u,h,s at $T_{r}=0.99$.

**Figure A7:**
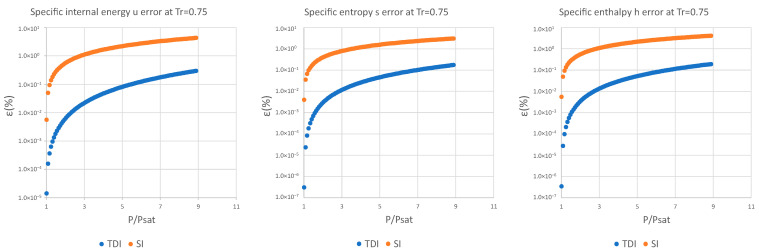
Relative errors for TDI and SI models in u,h,s at $T_{r}=0.75$.

**Figure A8:**
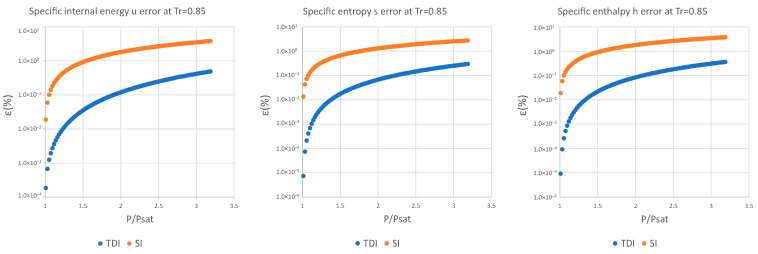
Relative errors for TDI and SI models in u,h,s at $T_{r}=0.85$.

**Figure A9:**
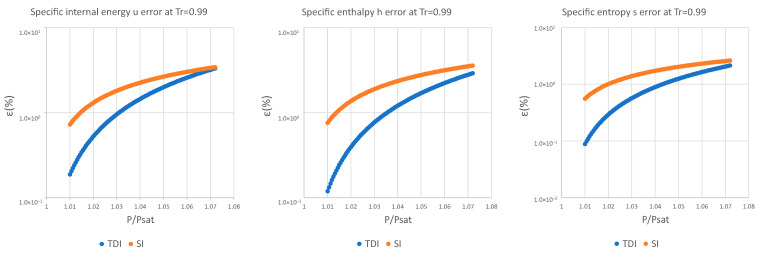
Relative errors for TDI and SI models in u,h,s at $T_{r}=0.99$.

## Data Availability

No new data were created or analyzed in this study. Data sharing is not applicable to this article.

## Appendix A

SUBTANCE: ISOBUTANE.

**Table A1:** Relative errors for TDI model for Isobutane.

$T_{r}$	$\varepsilon_{TDI.\;min.u}$ (%)	$\varepsilon_{TDI.\;max.u}$ (%)	$\varepsilon_{TDI.\;min.h}$ (%)	$\varepsilon_{TDI.\;max.h}$ (%)	$\varepsilon_{TDI.\;min.s}$ (%)	$\varepsilon_{TDI.\;max.s}$ (%)
0.3	1.94 × 10^−13^	1.25 × 10^−12^	3.48 × 10^−13^	1.14 × 10^−2^	1.90 × 10^−3^	9.48 × 10^−3^
0.5	8.82 × 10^−10^	5.63 × 10^−12^	6.96 × 10^−12^	7.72 × 10^−2^	2.87 × 10^−2^	3.67 × 10^−2^
0.6	5.68 × 10^−8^	6.56 × 10^−10^	6.91 × 10^−10^	6.06 × 10^−2^	3.09 × 10^−2^	3.42 × 10^−2^
0.75	5.84 × 10^−6^	1.47 × 10^−7^	1.25 × 10^−7^	1.04 × 10^−1^	6.98 × 10^−2^	6.12 × 10^−2^
0.9	3.60 × 10^−4^	3.00 × 10^−5^	2.19 × 10^−5^	3.13 × 10^−1^	2.51 × 10^−1^	1.85 × 10^−1^
0.99	9.14 × 10^−2^	5.83 × 10^−2^	4.02 × 10^−2^	1.55 × 10^0^	1.38 × 10^0^	9.48 × 10^−1^

SUBTANCE: R134A.

**Table A2:** Relative errors for TDI model for R134A.

$T_{r}$	$\varepsilon_{TDI.\;min.u}$ (%)	$\varepsilon_{TDI.\;max.u}$ (%)	$\varepsilon_{TDI.\;min.h}$ (%)	$\varepsilon_{TDI.\;max.h}$ (%)	$\varepsilon_{TDI.\;min.s}$ (%)	$\varepsilon_{TDI.\;max.s}$ (%)
0.5	3.10 × 10^−11^	6.33 × 10^−14^	1.94 × 10^−13^	1.82 × 10^−2^	7.56 × 10^−3^	1.21 × 10^−2^
0.6	7.61 × 10^−9^	9.06 × 10^−11^	1.04 × 10^−10^	2.55 × 10^−2^	1.36 × 10^−2^	1.61 × 10^−2^
0.75	1.98 × 10^−6^	5.04 × 10^−8^	4.33 × 10^−8^	6.13 × 10^−2^	4.22 × 10^−2^	3.70 × 10^−2^
0.9	1.99 × 10^−4^	1.62 × 10^−5^	1.14 × 10^−5^	2.28 × 10^−1^	1.85 × 10^−1^	1.32 × 10^−1^
0.99	5.89 × 10^−2^	3.70 × 10^−2^	2.43 × 10^−2^	1.25 × 10^0^	1.12 × 10^0^	7.35 × 10^−1^

SUBTANCE: CO_2_.

**Table A3:** Relative errors for TDI model for CO_2._

$T_{r}$	$\varepsilon_{TDI.\;min.u}$ (%)	$\varepsilon_{TDI.\;max.u}$ (%)	$\varepsilon_{TDI.\;min.h}$ (%)	$\varepsilon_{TDI.\;max.h}$ (%)	$\varepsilon_{TDI.\;min.s}$ (%)	$\varepsilon_{TDI.\;max.s}$ (%)
0.75	1.41 × 10^−5^	3.48 × 10^−7^	3.04 × 10^−7^	2.97 × 10^−1^	1.92 × 10^−1^	1.75 × 10^−1^
0.8	5.07 × 10^−5^	1.70 × 10^−6^	1.42 × 10^−6^	3.62 × 10^−1^	2.52 × 10^−1^	2.19 × 10^−1^
0.85	1.86 × 10^−4^	9.23 × 10^−6^	7.40 × 10^−6^	4.89 × 10^−1^	3.63 × 10^−1^	3.00 × 10^−1^
0.9	7.83 × 10^−4^	6.50 × 10^−5^	5.01 × 10^−5^	7.35 × 10^−1^	5.77 × 10^−1^	4.53 × 10^−1^
0.95	5.11 × 10^−3^	9.14 × 10^−4^	6.85 × 10^−4^	1.29 × 10^0^	1.07 × 10^0^	8.06 × 10^−1^
0.99	1.88 × 10^−1^	1.19 × 10^−1^	8.86 × 10^−2^	3.31 × 10^0^	2.91 × 10^0^	2.15 × 10^0^
